# Validation of the French Version of the “Patterns of Activity Measure” in Patients with Chronic Musculoskeletal Pain

**DOI:** 10.1155/2017/6570394

**Published:** 2017-02-01

**Authors:** Charles Benaim, Bertrand Léger, Philippe Vuistiner, François Luthi

**Affiliations:** ^1^Department of Physical Medicine and Rehabilitation, Orthopaedic Hospital, Lausanne University Hospital, Lausanne, Switzerland; ^2^Institute for Research in Rehabilitation, Clinique Romande de Réadaptation, Sion, Switzerland; ^3^Institute of Social and Preventive Medicine, Lausanne University Hospital, Lausanne, Switzerland; ^4^Department for Musculoskeletal Rehabilitation, Clinique Romande de Réadaptation, Sion, Switzerland

## Abstract

*Background*. The “Patterns of Activity Measure” (POAM-P) is a self-administered questionnaire that assesses “avoidance”, “pacing” and “overdoing” activity patterns in chronic pain patients.* Objectives*. To adapt the POAM-P to French (“POAM-P/F”) and test its validity and reliability in Chronic Musculo-Skeletal Pain patients (CMSP).* Methods*. We followed the recommended procedure for translation of questionnaires. Five hundred and ninety five inpatients, admitted to a tertiary rehab center in the French-speaking part of Switzerland for chronic pain after orthopedic trauma, were included (sex ratio M/F = 4.36, mean age 43 ± 12). Face, content and criterion validities, internal consistency and reliability were assessed. Data included: TAMPA Scale for Kinesiophobia (TSK), Chronic Pain Coping Inventory (CPCI), Pain Catastrophizing Scale (PCS), Brief Pain Inventory (BPI), Hospital Anxiety and Depression Scale (HADS).* Results*. Face and content validities were checked during the translation process. Correlations between POAM-P/F-avoidance and TSK, POAM-P/F-pacing and CPCI-pacing, POAM-P/F-overdoing and CPCI-task persistence were highly significant (*r* > 0.3, *p* < 10^−2^). The three subscales demonstrated excellent homogeneity (Cronbach's alpha coefficients > 0.8) and test-retest reliability (Intraclass Correlation Coefficients > 0.8). They correlated very differently with the other scales.* Discussion and Conclusion*. The three POAM-P/F subscales clearly assess different behaviors in CMSP. The POAM-P/F is a suitable questionnaire for classifying French speaking CMSP into avoiders, pacers or overdoers.

## 1. Introduction

Chronic musculoskeletal pain is one of the most frequent reasons for clinical encounters [1] and contributes to high indirect costs [2, 3], disability [4], and depressive mood [5]. There is increasing evidence that psychosocial factors are involved in the transition from acute to chronic pain, as well as with the maintenance of pain (e.g., see Helmus et al. [6]). Among them, fear beliefs, catastrophizing, and emotional distress seem to be the most important ones [7]. According to the “fear avoidance model,” pain patients with catastrophizing thoughts begin to worry and to avoid activities [8, 9]. This behavior leads to physical deconditioning, prevents recovery, and finally traps patients in a vicious cycle of chronic pain and disability. This model has been validated in many studies (for details see Leeuw et al. [10]). Besides “avoidance,” so-called “overdoing” behaviors (also named “endurance” or “persistence”) were also described [11]. Patients with “overdoing” behavior sustain physical activity despite pain and consequently reduce the normal recovery period with a higher risk of chronicity. This pattern seems to be beneficial in the short term but finally promotes the perpetuation of pain [12]. “Pacing” is a third activity pattern that was described when patients modulate their activity according to pain level (going slowly, taking breaks, etc.) [13]. “Pacing” seems to be the most adaptive strategy, but it remains unclear if it really is the most efficient in terms of pain reduction and functional outcome (see [14] or [15] for a structured review of the literature). Indeed, Kindermans et al. reported poor outcomes in a subgroup of “pacing” patients [16]. These results suggest that some “pacers” may adopt an inadequate strategy and may be in fact “hidden avoiders” [16]. Similarly, subgroups of “overdoers” have been identified. The first one called “functional performers” demonstrates a high activity level despite pain [17]. The second subgroup of patients that can be characterized as “mixed performers” may alternate between overdoing and avoidance behaviors [18]. In summary, if activity “avoidance” seems generally to be related to poor outcomes, the situation appears to be more complex for “overdoing” or “pacing” behaviors.

The “Patterns of Activity Measure” (POAM-P) is a self-administered questionnaire which aims to assess “avoidance,” “pacing,” and “overdoing” activity patterns [19]. The original English POAM-P showed good psychometric properties in patients referred to an interdisciplinary pain management program for chronic pain [19]. Its usability was excellent: less than 2 patients out of 166 were excluded from the analysis because of missing values. The three subscales of the POAM-P demonstrated excellent internal consistency; responses to the questionnaires were only marginally influenced by social desirability. In the same study, Cane et al. showed that pacing was associated with positive outcomes whereas avoidance and overdoing were associated with negative psychosocial outcomes. The Dutch version of the POAM-P was successfully adapted and validated by Kindermans et al. [20]. The 3-factor structure was confirmed and the subscales were found to be highly internally consistent. Substantial correlations were found with fear, catastrophizing, depressive symptoms, and disability. Huijnen et al. then investigated the relationship between the Dutch version of the POAM-P avoidance and overdoing subscales and both an objectively assessed and a subjectively reported measure of physical activity in a group of low back pain patients [17]. Using a combination of the median scores of avoidance and overdoing subscales, he proposed 4 different activity-related behaviors: avoiders, persisters, functional performers (low scores in both avoidance and persistence behaviors), and mixed performers (high scores in both avoidance and persistence behaviors). Self-reported disability was higher in avoiders, persisters, and mixed performers. Avoiders also showed a low level of self-reported daily activities whereas persisters were characterized by objectively measured longer daily physical activity. Nevertheless the objectively assessed activity level was not statistically different between the 4 groups, and there was no association between objective activity level and pain intensity [17]. More recently, a Spanish version of the POAM-P was translated and used to develop the Activity Patterns Scale [21]. The above-mentioned studies strongly suggest that there is a real need for additional work which aims to characterize activity patterns in different models of CMSP patients. It is also of prime importance to validate a tool that may take into account other cultural backgrounds than English or Dutch native speakers, which represent the most investigated populations so far. Our objective was to adapt the POAM-P to French (“POAM-P/F”) and to test the validity and reliability of the French version in patients with chronic musculoskeletal pain (CMSP).

## 2. Methods

A single-center prospective study was conducted in a tertiary rehab center, in the French-speaking part of Switzerland. All rehab inpatients of working age, admitted for CMSP after orthopedic trauma between May 2013 and July 2015, were considered for inclusion. Inclusion criteria were as follows: French-speaking working-age patients, patients able to understand and sign the study informed consent form, patients able to answer the questionnaires used in the study, and patients suffering from CMSP (at least 6 months). Patients were excluded from the study if they met any of the following criteria: severe traumatic brain injury at the time of the accident (Glasgow coma scale ≤ 8), spinal cord injury, and being under legal custody. Data collected included demographic variables (sex, age), pain location (neck/back, upper limb, and lower limb), and the following questionnaires: POAM-P/F, TAMPA Scale for Kinesiophobia (TSK) [8], “pacing” and “task persistence” subscales of the Chronic Pain Coping Inventory (CPCI) [13, 22], Pain Catastrophizing Scale (PCS) [23], Brief Pain Inventory (BPI) [24], and Hospital Anxiety and Depression Scale (HADS) [25].

The POAM-P is composed of thirty questions, 10 for each pattern (see Annex I in Supplementary Material available online at https://doi.org/10.1155/2017/6570394). For each question, patients are asked to describe to which extent the selected item describes how they usually perform their activity using a scale ranging from 0 “not at all” to 4 “all the time.” Thus, the score ranges from 0 to 40 for each pattern of behavior and the patient's pattern of behavior is classified as “avoidance,” “pacing,” or “overdoing” according to the highest score (no cut-off score is proposed).

### 2.1. Translation

The validation of the POAM-P/F (see Annex  II) followed the recommended procedure for translation and transcultural adaptation of questionnaires [26]. (1) The POAM-P was first independently translated from English to French by two bilingual translators: one health professional (psychologist) and one linguist. The two resulting versions of the POAM-P/F were examined by a six-member bilingual committee composed of two linguists, two physicians, and two scientists. Minor differences between the two French versions were noticed, like: “*quand je fais une activité*”* versus* “*quand je suis en train de faire une activité.*” A consensus was reached, leading to a first consensual version of the POAM-P/F; (2) two back translations (French to English) of this first intermediate version were carried out independently by two bilingual linguists. Then the same six-member expert committee compared these back translations with the original POAM-P in terms of conceptual and semantic equivalence and made minor modifications to the first POAM-P/F version, leading to a second French version; (3) pretesting is as follows: this second intermediate version was applied to a group of twenty CMSP patients (sex ratio M/F 2.33, mean age 45 ± 11, and range 24–61) in order to verify that the items were clearly understood. As the patients' feedback was satisfactory, no change was made to the POAM-P/F at this stage.

### 2.2. Validation

Face and content validities were assessed during the transcultural validation process of the questionnaire, that is to say, consistency, representativeness and clarity of the POAM-P/F items. Five hundred and ninety-five patients (sex ratio M/F 4.36, mean age 43 ± 12, and range 18–69) participated in the following steps: (1) criterion validity of POAM-P/F “avoidance” was assessed by its correlation to the TSK; POAM-P/F “pacing” and “overdoing” subscores were compared to the corresponding subscores of the CPCI. As the French version of the CPCI was available and implemented in our clinic later compared to the TSK, inclusions were stopped when the number of completed CPCI scales reached the number of subjects required. This number was estimated to be 82, giving the following parameters: *r*(H0) = 0, *r*(H1) = 0.35 (based on preliminary unpublished data), power = 0.8, and *α* = 0.05. Finally, 94 patients underwent the CPCI questionnaire; (2) internal consistency was evaluated in the whole group of CMSP patients; (3) test-retest reliability was assessed in a subgroup of 83 patients who completed the POAM-P/F twice in a 2-week period. The number of subjects required for this step was estimated to be 51, giving an expected Intraclass Coefficient Correlation of 0.8 [0.7–0.9]_95%_.

The study was approved by the local ethics committee (CCVEM 034/12). All participants signed an informed consent form before enrolment.

### 2.3. Data Analyses

All statistical analyses were performed using NCSS 9 [27]. Criterion validity was evaluated by (1) the Pearson correlation coefficient between the POAM-P/F scores of “avoidance,” “pacing,” and “overdoing” subscales and the TSK score (all patients) and the corresponding CPCI subscores (94 patients), respectively, and (2) a Principal Component Analysis (PCA) run with POAMP-P/F avoidance, POAMP-P/F pacing, POAMP-P/F overdoing, TSK, HASD-anxiety, HADS-depression, PCS, BPI-severity, BPI-interference, age, and number of surgeries. Internal consistency of the three subscales “avoidance,” “pacing,” and “overdoing” was assessed with the Cronbach's alpha coefficient. Test-retest reliability was assessed with the Intraclass Correlation Coefficient (ICC).

## 3. Results

Among the five hundred and ninety-five patients, median pain duration was 407 days (90 days–32 years). The pain was located in the lower limb in 249 patients (42%), in the upper limb in 232 patients (39%), in the neck/back in 94 patients (16%), and in more than one location in 20 patients (3%). Two hundred and eighty-nine patients (49%) were classified as avoiders by the POAM-P/F, 160 (27%) as pacers, and 129 (22%) as overdoers, and 17 (3%) could not be classified (identical scores in the two best-scored subscales). One hundred and eighty patients (30%) had one surgery and 208 (35%) had more than one. In two hundred and sixty-seven patients (46%), the initial pain was due to a workplace accident. Fifty-five patients (9%) were involved in litigation against their insurance company. Scores for the questionnaires are reported in Table 1. Men had slightly higher avoidance scores than women (28.8 ± 8.4 versus 27.1 ± 8.6) and slightly lower overdoing scores (20.7 ± 8.7 versus 22.3 ± 9.3), with a substantial trend toward significance (*p* = 0.06 and 0.07, resp.). Pacing scores were close between men and women (26.2 ± 8.2 versus 25.3 ± 10.3, *p* = 0.32). TSK scores were significantly higher for men (45.2 ± 7.8 for men versus 41.6 ± 8.4 for women, *p* < 10^−4^). Age correlated with pacing (*r* = 0.246, *p* < 10^−4^), but not with avoidance and overdoing (*r* = 0.061, *p* = 0.14 and *r* = −0.067, *p* = 0.10, resp.).

### 3.1. Translation

The final version of the POAM-P/F is presented in Annex II. The POAM-P/F was easy to administer and all patients from the pretesting group completed the whole questionnaire. After each completion of the questionnaire, patients were solicited to providing a feedback on the questionnaire. The most frequent comment was regarding redundancy between some questions (12 out of 20 patients). The usability of the questionnaire has been assessed as “good” or “very good” by all the patients.

### 3.2. Validation

#### 3.2.1. Criterion Validity

The correlation between the “avoidance” score of the POAM-P/F and TSK was highly significant (*r* = 0.508, *p* < 10^−4^; see Table 1), as well as the correlation between the “pacing” and “overdoing” scores of the POAM-P/F and the corresponding CPCI subscores (*r* = 0.307, *p* < 10^−2^, and *r* = 0.312, *p* < 10^−2^). As expected, avoidance correlated negatively with overdoing (*r* = −0.370, *p* < 10^−4^). The correlation between overdoing and pacing was also negative (*r* = −0.160, *p* = 10^−4^). Avoidance was correlated to all other scores, except CPCI-pacing. Six independent factors from the PCA with varimax rotation included 83% of the total variation (Table 2). Factor (1) was highly correlated to HADS-anxiety, HADS-depression, and PCS, Factor (2) with avoidance and pacing, Factor (3) with BPI-severity and BPI-interference, Factor (4) with age, Factor (5) with the number of surgeries, and Factor (6) with overdoing. Thus, these independent factors could be interpreted as follows: (1) mood, (2) avoidance/pacing, (3) pain, (4) age, (5) surgery, and (6) overdoing. Aside from these very strong correlations, we noticed that avoidance slightly correlated with Factor (1) (same direction as psychological scales), Factor (3) (same direction as pain scales), and Factor (6) (opposite direction to overdoing); pacing slightly correlated with Factor (4) (same direction as age). None of the three behavioral patterns participated towards Factor (5) (surgery). TSK correlated with both Factors (1) (same direction as psychological scales) and (2) (same direction as avoidance and pacing).

#### 3.2.2. Reliability

ICC were 0.881 [0.821–0.921]_95%_ for avoidance, 0.865 [0.799–0.911]_95%_ for pacing, and 0.731 [0.613–0.818]_95%_ for overdoing. Cronbach's alpha coefficients were 0.877, 0.891, and 0.846 for avoidance, pacing, and overdoing subscales, respectively.

## 4. Discussion

Psychological assessment is a key point of a rehabilitation program in chronic pain patients. With this aim in view, questionnaires such as the HADS, TSK, and POAM-P are useful to assess mood/anxiety and patterns of behavior. Whereas the TSK is specifically designed to detect avoiders, the POAM-P aims at classifying patients into three categories: avoiders, pacers, and overdoers. At the moment, there is considerable debate on the classification of behavior patterns and on their implications in the management of patients [14, 17, 18, 28]. Nonetheless, standardized questionnaires such as the POAM-P are an integral part of the evaluation of chronic pain patients in the musculoskeletal field, notably for research purposes.

In this study, the original English version of POAM-P was translated and adapted into French, to create the POAM-P/F. Criterion validity and reliability of the POAM-P/F were satisfactory and supported the use of this questionnaire to determine behavior patterns in patients with CMSP. The POAM-P/F is easy to administer (it takes only a few minutes), valid, and reliable, the latter property having never been assessed with the original English version. The avoidance, pacing, and overdoing subscales of the POAM-P/F demonstrated excellent internal consistency. A small number of patients (3% in the present study) could not be classified by the POAM-P/F when two subscales had the same highest scores, for example, “avoidance 30 - pacing 30 - overdoing 20”. In such cases the TSK score may be used to confirm or reverse avoidance behavior. However, other cases such as “avoidance 20 - pacing 30 - overdoing 30” cannot be resolved this way. In this case the behavior classification could alternatively rely upon the “median score” rule (above or below), as suggested by Huijnen et al. [17], but this has the disadvantage of being an* a posteriori* analysis with varying cut-off scores depending on the sample. Determining meaningful cut-off scores of the POAM-P subscales is still needed. Patients from the pretest group noticed that there is some redundancy among certain questions of the POAM-P/F. This of course was not a surprise since it is also the case in most of the questionnaires currently used. A small dose of redundancy in a questionnaire allows better measuring of what it is intended to. It also can be useful for detecting and quantifying unreliability in respondents, like in the Hand Function Sort [29]. Similarly, repeated clinical testing can give more reliable results. However, the proliferation of standardized clinical tests or questionnaires increases the burden for examiner and respondent. Currently, some patients, especially those with low educational level [30], may need more than one hour to complete a whole battery of questionnaires used in most clinical studies. This also limits the use of questionnaires in daily practice. This topic has been thoroughly discussed in the literature (see Fokkema et al. 2014 for application in psychological measurements) [31] and the use of short-form versions of questionnaires has to be considered. As in the original English version of the POAM-P [19], interscale correlations were all significant. As expected, the highest negative correlation was found between avoidance and overdoing. Conversely, the highest positive correlation was found between avoidance and pacing. This result confirmed previous works suggesting that some pacers could be in reality “hidden avoiders” [16]. Our results can be summarized as follows: (1) avoidance and overdoing are more or less opposite patterns, higher pain intensity and psychological disturbances going with higher avoidance scores and lower overdoing scores; (2) pacing is close to avoidance, but whereas avoidance is linked to pain intensity and psychological disturbances, pacing is linked to higher age. Therefore, these three sets of items clearly assess three different behaviors in CMSP patients. As previously demonstrated by Cane et al., the correlation between avoidance items and the TSK score was high, suggesting that the criterion validity of these items was good. The same result was found for pacing and overdoing scores that were significantly correlated to the corresponding CPCI subscores. Higher scores of pacing were observed in older patients. This finding is in line with Molton et al.'s work [32] that assessed patients with chronic pain associated with neurological impairments. In this study, older adults tended to use a wider range of strategies than younger adults did, especially “activity pacing,” “seeking social support,” and “coping self-statements.” As in previous works [18, 19], we observed more avoiders in men and more overdoers in women. However, these differences failed to be statistically significant, despite the size of our cohort. This lack of significance was possibly due to a much higher sex ratio M/F in our working-age population than in previous cohorts (4.36 versus 0.26 in Cane et al. [19] and 0.34 in McCracken and Samuel [18]). Since the present study was not an epidemiological study, this selection bias is not a major drawback. The independent PCA factors could be interpreted as follows (Factors (1) to (6)): (1) mood, (2) pain, (3) avoidance/pacing, (4) age, (5) overdoing, and (6) number of surgical operations. To a lesser degree, Factors (1) and (3) could also be identified as kinesiophobia factors. Of course this does not mean that mood, avoidance, and pain were not correlated at all. This apparent independence is simply due to the rotation option used in the PCA. The “varimax” rotation usually yields results which make it as easy as possible to identify each variable with a single factor, and this is why it is the most popular rotation option. However, the orthogonality (i.e., independence) of factors is often an unrealistic assumption [33].

As all of our patients were of working age and suffering from CMSP after orthopedic trauma, the main limitation of this study is the selection bias. Most patients were men, aged 18–69, and manual workers. One could imagine that patterns, psychological, or pain scores would have been slightly different in a group of patients suffering from CMSP of any origin. However, as for age and sex (already discussed above), this is not a major drawback in a questionnaire's transcultural validation process.

There is another potential bias in the present study, as in the vast majority of health and social surveys: we have no idea if patients respond in accordance to how they truly feel or to social norms. This phenomenon is called “social desirability” (SD) and can question the validity of questionnaires assessing psychological status, pain, or disability. In Deshields et al.'s work on chronic pain [34], patients with higher scores on the Marlowe-Crowne Social Desirability Scale (MC-SDS) [35] reported less depression and anxiety but higher levels of pain severity. Knowing this, should we systematically include such a questionnaire in our battery of tests? And if we do so, should we consider as worthless data collected in patients with higher scores of SD? In clinical research, data anonymization is supposed to reduce this kind of bias, but it is not the case in clinical practice. In the POAM-P/F, people are asked how they manage their activity level according to their pain. Patients with high SD could answer according to what they consider to be a reasonable behavior rather than to what they actually do. This could partially explain why some patients classified as “pacers” are suspected to be in reality “hidden avoiders” and have a bad functional outcome [16]. In a previous work, Cane et al. demonstrated that responses to the POAM-P were not significantly affected by SD [19]. However, as mentioned before, this does not seem to be the case for other questionnaires (e.g., pain, depression, and anxiety) used for research on patterns of behavior [34]. For these, correlation with SD should therefore be systematically assessed. These considerations are not of great importance in a transcultural validation study, but future research aiming at refining the classification of patterns of behaviors should address this issue. With this aim in view, one should consider the acceptability of SD questionnaires in CMSP patients. Indeed, we have no idea how patients could react to questions that have nothing to do with their pathology, as in the MC-SDS: “*Before voting I thoroughly investigate the qualifications of all the Candidates*” or “*I am always careful about my manner of dress.*” Short-form versions of SD questionnaires should probably be a preferable option.

To conclude, this study demonstrated that the POAM-P/F is a suitable questionnaire for classifying CMSP patients into avoiders, pacers, or overdoers and that its psychometric properties are close to those of its original English version. This new version will also promote studies in new populations of pain patients with different cultural backgrounds. Avoidance, pacing, and overdoing seem to be three very different patterns, but further studies are needed to clarify and probably refine this classification before attempting to tailor a rehabilitation program according to patients' patterns of behavior.

## Supplementary Material

The POAM-P is composed of thirty questions, 10 for each pattern. For each question, patients are asked to describe to which extent the selected item describes how they usually perform their activity using a scale ranging from 0 “not at all” to 4 “all the time.” Thus, the score ranges from 0 to 40 for each pattern of behavior and the patient's pattern of behavior is classified as “avoidance,” “pacing,” or “overdoing” according to the highest score.

## Figures and Tables

**Table 1:** Mean scores for questionnaires/assessments and correlations with POAM-P/F subscores.

Questionnaire/assessment	Mean ± SD	Min–max	Correlation with POAM-P/F avoidance	Correlation with POAM-P/F pacing	Correlation with POAM-P/F overdoing
POAM-P/F avoidance (0–40)	28 ± 8	0–40	—	—	—
POAM-P/F pacing (0–40)	26 ± 9	0–40	*r* = 0.480, *p* < 10^−4^	—	—
POAM-P/F overdoing (0–40)	21 ± 9	0–39	*r* = −0.370, *p* < 10^−4^	*r* = −0.160, *p* < 10^−4^	—
TSK (17–68)	45 ± 8	24–63	*r* = 0.508, *p* < 10^−4^	*r* = 0.218, *p* < 10^−4^	*r* = −0.289, *p* < 10^−4^
CPCI pacing (0–7)	3.4 ± 1.9	0–7	*r* = −0.140, *p* = 0.18	*r* = 0.307, *p* < 10^−2^	*r* = 0.233, *p* = 0.02
CPCI overdoing (0–7)	2.6 ± 2.0	0–7	*r* = −0.393, *p* < 10^−4^	*r* = −0.208, *p* = 0.04	*r* = 0.312, *p* < 10^−2^
HADS-anxiety (0–21)	9.3 ± 4.3	0–20	*r* = 0.228, *p* < 10^−4^	*r* = 0.004, *p* = 0.93	*r* = −0.141, *p* < 10^−3^
HADS-depression (0–21)	7.1 ± 4.2	0–20	*r* = 0.251, *p* < 10^−4^	*r* = 0.029, *p* = 0.49	*r* = −0.267, *p* < 10^−4^
PCS (0–52)	23 ± 12	0–52	*r* = 0.428, *p* < 10^−4^	*r* = 0.134, *p* = 10^−3^	*r* = −0.276, *p* < 10^−4^
BPI-severity (0–10)	4.5 ± 2.0	0–9.3	*r* = 0.338, *p* < 10^−4^	*r* = 0.133, *p* = 10^−3^	*r* = −0.281, *p* < 10^−4^
BPI-interference (0–10)	4.6 ± 2.2	0–10	*r* = 0.318, *p* < 10^−4^	*r* = 0.098, *p* = 0.02	*r* = −0.201, *p* < 10^−4^

**Table 2:** Factor loadings of the Principal Components Analysis.

Variables	Factor (1) 23%	Factor (2) 16%	Factor (3) 15%	Factor (4) 10%	Factor (5) 10%	Factor (6) 9%
Avoidance	−0.191	–**0.781**	0.212	−0.075	−0.052	−0.264
Pacing	0.066	*– * **0.841**	0.034	0.280	−0.014	0.051
Overdoing	0.126	0.158	−0.126	−0.050	0.005	**0.953**
TSK	−0.535	−0.533	0.094	−0.196	−0.044	−0.189
HADS-anxiety	−**0.875**	−0.001	0.189	0.029	−0.002	0.034
HADS-depression	−**0.852**	−0.004	0.183	0.021	0.048	−0.146
PCS	−**0.740**	−0.253	0.363	−0.056	−0.076	−0.109
BPI-severity	−0.201	−0.131	**0.871**	0.045	−0.065	−0.156
BPI-interference	−0.392	−0.099	**0.795**	0.009	0.019	−0.010
Age	0.008	−0.097	0.039	**0.961**	−0.051	−0.046
Nb of surgery	0.003	0.051	−0.038	−0.050	**0.994**	0.008

## Additional Points

*Summary*. The “Patterns of Activity Measure” (POAM-P) is a self-administered questionnaire which aims to assess “avoidance,” “pacing,” and “overdoing” activity patterns in individuals with chronic pain. We translated the POAM-P into French and checked the validity and reliability of its French version. Our study on 595 patients demonstrated that (1) avoidance and overdoing are more or less opposite patterns, higher pain intensity and psychological disturbances going with higher avoidance scores and lower overdoing scores and that (2) pacing is close to avoidance, but whereas avoidance is linked to higher pain intensity and psychological disturbances, pacing is linked to higher age.
