# Supplementary material for: Validation of the French Version of the “Patterns of Activity Measure” in Patients with Chronic Musculoskeletal Pain
Source: Pain Res Manag. 2017 Feb 1;2017:6570394. doi: 10.1155/2017/6570394 (PMC5309399; doi:10.1155/2017/6570394)
Supplement: Supplementary file 1 — The POAM-P is composed of thirty questions, 10 for each pattern. For each question, patients are asked to describe to which extent the selected item describes how they usually perform their activity using a scale ranging from 0 “not at all” to 4 “all the time.” Thus, the score ranges from 0 to 40 for each pattern of behavior and the patient's pattern of behavior is classified as “avoidance,” “pacing,” or “overdoing” according to the highest score. [file 6570394.f1.docx]

**ANNEX I: The original English POAM-P**

Avoidance: Items 1, 6, 8, 11, 13, 16, 19, 22, 25, 28

Pacing: Items 3, 5, 9, 12, 14, 17, 21, 24, 27, 29

Overdoing: Items 2, 4, 7, 10, 15, 18, 20, 23, 26, 30

1. I stop what I am doing when my pain starts to get worse.

2. When I’m doing an activity I don’t stop until it is finished.

3. I go back and forth between working and taking breaks when doing an activity.

4. I take on extra tasks when I am having a good pain day.

5. When I start an activity I think about how to split it into smaller parts.

6. There are many activities that I avoid because they flare up my pain.

7. I make the most of my good pain days by doing more things.

8. When my pain starts to get worse I know it’s time to stop what I am doing.

9. I do my activities at a slow and steady pace.

10. I keep doing what I am doing until my pain is so bad that I have to stop.

11. I avoid activities that I know will make my pain worse.

12. When I do an activity I stop after a while and then come back later to do more.

13. Most days my pain keeps me from doing much at all.

14. I go slower and work at a steady pace when I’m doing things.

15. Once I start an activity I keep going until it is done.

16. I limit my activities to the ones that I know will not make my pain worse.

17. When I do an activity I break it into small parts and do one part at a time.

18. I just ignore my pain and keep doing what I’m doing as long as I can.

19. Because of my pain most days I spend more time resting than doing activities.

20. I keep going until I can’t stand the pain anymore.

21. Instead of doing an activity all at once I do a little bit at a time.

22. I don’t start an activity if I know it will make my pain worse.

23. I do extra on days when my pain is less.

24. I remember to stop and take breaks when I’m doing an activity.

25. If I know that something will make my pain worse I don’t do it anymore.

26. When I do an activity I do the whole thing all at once.

27. Instead of doing the whole activity I divide it into small parts and do one part at a time.

28. I’ve cut back my activities by not doing the ones that make my pain worse.

29. When I do an activity I work for a while, take a break, and then go back to work again.

30. Some days I do a lot, other days I don’t do much.

**ANNEX II: The POAM-P/F**

Evitant : Items 1, 6, 8, 11, 13, 16, 19, 22, 25, 28

Modulant : Items 3, 5, 9, 12, 14, 17, 21, 24, 27, 29

Persistant : Items 2, 4, 7, 10, 15, 18, 20, 23, 26, 30

1. J’arrête ce que je suis en train de faire quand la douleur commence à empirer

2. Quand je fais une activité, je ne m’arrête pas jusqu’à ce qu’elle soit terminée

3. Quand je fais quelque chose, j’alterne les moments d’activité et de pause

4. Les bons jours sans trop de douleur, je fais davantage de choses

5. Quand je commence une activité, je réfléchis à la façon de la découper en plus petites tâches

6. Il y a beaucoup d’activités que j’évite parce qu’elles réveillent la douleur

7. Je profite au maximum des bons jours sans trop de douleur pour faire davantage de choses

8. Quand la douleur commence à empirer, je sais qu’il est temps d’arrêter ce que je suis en train de faire

9. Je fais mes activités à un rythme lent et régulier

10. Je continue de faire ce que je suis en train de faire jusqu’à ce que la douleur soit si forte que je dois m’arrêter

11. J’évite les activités dont je sais qu’elles feront empirer la douleur

12. Quand je fais une activité, j’arrête après un moment et je la reprends un peu plus tard

13. La plupart du temps, la douleur m’empêche de faire beaucoup de choses

14. Je vais plus lentement et je travaille à un rythme régulier quand je fais quelque chose

15. Une fois que j’ai commencé une activité, je la continue jusqu’à ce qu’elle soit terminée

16. Je limite mes activités à celles dont je sais qu’elles ne feront pas empirer la douleur

17. Quand je fais une activité, je la divise en petites tâches et je fais une chose après l’autre

18. J’ignore simplement la douleur et je continue de faire ce que je suis en train de faire aussi longtemps que je le peux

19. A cause de la douleur, je passe la plupart des jours à me reposer plutôt qu’à faire des activités

20. Je continue ce que je suis en train de faire jusqu’à ce que je ne puisse plus supporter la douleur

21. Au lieu de faire une activité en une fois, je fais un petit peu à la fois

22. Je ne commence pas une activité si je sais qu’elle fera empirer la douleur

23. Je fais davantage les jours où la douleur est moins forte

24. Je n’oublie pas de m’arrêter et de faire des pauses quand je fais une activité

25. Si je sais que quelque chose fera empirer la douleur, je ne le fais plus

26. Quand je fais une activité, je la fais en une seule fois

27. Au lieu de faire toute l’activité en une fois, je la divise en petites tâches et je fais une chose à la fois

28. J’ai diminué mes activités en ne faisant pas celles qui font empirer la douleur

29. Quand je fais une activité, je travaille pendant un moment, je fais une pause, puis je reprends mon activité

30. Certains jours, je fais beaucoup, d’autres jours je fais peu
